# Association of non-HLA antibodies against endothelial targets and donor-specific HLA antibodies with antibody-mediated rejection and graft function in pediatric kidney transplant recipients

**DOI:** 10.1007/s00467-021-04969-1

**Published:** 2021-03-24

**Authors:** Alexander Fichtner, Caner Süsal, Britta Höcker, Susanne Rieger, Rüdiger Waldherr, Jens H Westhoff, Anja Sander, Duska Dragun, Burkhard Tönshoff

**Affiliations:** 1 Department of Pediatrics I, University Children’s Hospital Heidelberg, Im Neuenheimer Feld 430, D-69120 Heidelberg, Germany; 2 Transplantation Immunology, Institute of Immunology, University Hospital Heidelberg, Im Neuenheimer Feld 305, D-69120 Heidelberg, Germany; 3 Department of Pathology, University Hospital Heidelberg, Im Neuenheimer Feld 224, D-69120 Heidelberg, Germany; 4 Institute of Medical Biometry and Informatics, University of Heidelberg, Im Neuenheimer Feld 305, D-69120 Heidelberg, Germany; 5 Clinic for Nephrology and Critical Care Medicine, Charite-Universitatsmedizin Berlin, Corporate member of Freie Universitat Berlin, Humboldt-Universitat zu Berlin, Berlin, Germany; 6 Berlin Institute of Health, Berlin, Germany

**Keywords:** Non-HLA antibodies, Endothelial targets, Donor-specific HLA antibodies, Antibody-mediated rejection, Graft function, Pediatric kidney transplant recipients

## Abstract

**Background:**

Non-HLA antibodies against endothelial targets have been implicated in the pathogenesis of antibody-mediated rejection (ABMR), but data in pediatric patients are scarce.

**Methods:**

We retrospectively analyzed a carefully phenotyped single-center (University Children’s Hospital Heidelberg, Germany) cohort of 62 pediatric kidney transplant recipients (mean age at transplantation, 8.6 ± 5.0 years) at increased risk of graft function deterioration. Patients had received their transplant between January 1, 1999, and January 31, 2010. We examined at time of late index biopsies (more than 1-year post-transplant, occurring after January 2004) the association of antibodies against the angiotensin II type 1 receptor (AT_1_R), the endothelin type A receptor (ET_A_R), the MHC class I chain-like gene A (MICA), and vimentin in conjunction with overall and complement-binding donor-specific HLA antibodies (HLA-DSA) with graft histology and function.

**Results:**

We observed a high prevalence (62.9%) of non-HLA antibody positivity. Seventy-two percent of HLA-DSA positive patients showed additional positivity for at least one non-HLA antibody. Antibodies against AT_1_R, ET_A_R, and MICA were associated with the histological phenotype of ABMR. The cumulative load of HLA-DSA and non-HLA antibodies in circulation was related to the degree of microinflammation in peritubular capillaries. Non-HLA antibody positivity was an independent non-invasive risk factor for graft function deterioration (adjusted hazard ratio 6.38, 95% CI, 2.11–19.3).

**Conclusions:**

Our data indicate that the combined detection of antibodies to HLA and non-HLA targets may allow a more comprehensive assessment of the patients’ immune responses against the kidney allograft and facilitates immunological risk stratification.

**Supplementary Information:**

The online version contains supplementary material available at 10.1007/s00467-021-04969-1.

## Introduction

Antibody-mediated rejection (ABMR) is the major cause of graft loss in both adult [1–3] and pediatric kidney transplant recipients [3, 4]. The majority of these rejections are caused by preformed and/or de novo donor-specific antibodies against human leucocyte antigens (HLA-DSA). However, there is a significant subset of patients with histological features of ABMR in the graft biopsy, in whom HLA-DSA cannot be detected in the circulation [5, 6]. In recent years, therefore, there have been increasing efforts directed towards the detection and biological characterization of antibodies against other endothelial targets beside HLA. For over a decade, investigation of non-HLA-specific antibodies and their impact on antibody injury and graft outcome has focused on antigens expressed by the vascular endothelium. Included in these antigens are the G protein coupled receptors (GPCRs) angiotensin type 1 receptor (AT_1_R) and endothelin type A receptor (ET_A_R) [7–16]. Antibodies against AT_1_R and ET_A_R have been detected in the sera of transplant recipients who experience allograft dysfunction [17]. Importantly, AT_1_R-Ab and ET_A_R-Ab activate their target receptors and are therefore considered not only biomarkers but potential contributors to allograft injury [7]. In addition, antibodies against MHC class I chain-like gene A (MICA) have been implicated in the rejection of kidney allografts [18–20]. MICA antigens are surface glycoproteins with functions related to innate immunity [21, 22]. MICA antigens are expressed on endothelial cells, dendritic cells, fibroblasts, epithelial cells, and many tumors, but not on peripheral-blood lymphocytes. As with the HLA antigens, which MICA antigens resemble only remotely in terms of structure, exposure to allogeneic MICA during transplantation can elicit antibody formation [23]. Other non-HLA antibodies are directed against a variety of proteins expressed by stressed endothelial cells including vimentin [24].

However, there are limited data available that correlate the presence of these antibodies with the histological phenotype of ABMR and the functional outcome of the graft. Especially in the vulnerable patient population of pediatric kidney allograft recipients, in whom, in the light of their long life expectancy, preservation of good graft function is of utmost importance, data on non-HLA antibodies are limited to AT_1_R-Ab [25–27]. The aims of the present study were therefore to investigate in a carefully phenotyped cohort of pediatric kidney transplant recipients at increased risk of graft deterioration (i) the prevalence of non-HLA antibodies in relation to HLA-DSA, (ii) the relationship of non-HLA and HLA antibodies with graft histology, and (iii) the role of non-HLA and HLA antibodies as risk factors for graft function deterioration.

## Patients and methods

### Study design and patient population

All non-presensitized pediatric patients aged ≤ 18 years at date of transplantation were eligible for this retrospective single-center consecutive cohort analysis based on prospectively collected serum samples. Because this study focused on the association of different antibody entities with graft histology, especially ABMR, and graft function development in the long-term, only patients with at least one indication biopsy more than 1-year post-transplant (late indication biopsy) were included in this study. As shown previously, late indication biopsies show a higher rate of ABMR than early indication biopsies [1]. In our institution (University Children’s Hospital Heidelberg), the post-transplant surveillance does not include regular protocol biopsies. In patients with sequential late indication biopsies, the “index biopsy” was defined as the first biopsy with a concomitant positive HLA-DSA serum sample. For HLA-DSA negative patients with sequential late indication biopsies, the “index biopsy” was defined as the one which matched best according to the time period from transplantation to biopsy with the index biopsy in HLA-DSA positive patients. Only patients with late indication biopsies after January 2004 were included, when routine C4d staining of biopsy specimens was introduced in our institution. All graft biopsies were re-evaluated by the use of the Banff 2015 classification [28]. Further inclusion and exclusion criteria were previously published elsewhere [25, 29]. In all, after exclusion of eight patients, 62 patients were included in the final data set (Supplementary Fig. 1).

Patient consent and ethics committee approval were obtained, and the investigations were performed in accordance with the Declaration of Helsinki and Good Clinical Practice (GCP) guidelines. Written informed consent was obtained from all parents or guardians and patients when appropriate for their age. This study was designed, analysed and reported according to the Strengthening the Reporting of Observational Studies in Epidemiology (STROBE) guidelines [30].

### Detection of HLA-DSA and non-HLA antibodies

HLA-DSA were measured concomitantly to any late indication biopsy. C1q-fixing HLA-DSA as well as five different non-HLA antibodies were retrospectively analyzed in serum samples taken at the time of index biopsy. Serum was collected in the framework of the ongoing prospective Collaborative Transplant Study (CTS) Serum Project. Analysis on C1q-fixing HLA-DSA and AT_1_R-Ab in this cohort were based on data captured and published previously [25, 29]; hence, Supplementary Table 1 was mostly replicated and only partly modified from Reference 25.

Patients’ sera were tested for HLA antibodies using LABScreen single antigen bead kits (One Lambda, Canoga Park, CA, USA). For high-resolution typing, CTS-PCR-SSP Tray and CTS-Sequence kits (Heidelberg, Germany), and Olerup SSP kits (Saltsjöbaden, Sweden) were used. A mean fluorescence intensity (MFI) of ≥ 500 was considered positive [31]. For the measurement of AT_1_R or ET_A_R antibodies, a sandwich ELISA (CellTrend GmbH, Luckenwalde, Germany, now One Lambda, Thermo Fisher Scientific, Canoga Park, CA, USA) was used. The inter-assay variability was 8%, and the intra-assay variability 5%. For both antibodies, a clinically predictive positive cutoff value of ≥ 10 U/mL was taken in accordance with the manufacturer’s instructions. Antibodies against MICA and human neutrophil antigen (HNA)-1 to -5 were measured using the single antigen bead kits of One Lambda (Canoga Park, CA, USA). In the case of MICA antibodies, an MFI value of ≥ 500 and in the case of HNA antibodies a ratio of ≥ 5 was defined as positive. Sera were analyzed for the presence of anti-vimentin antibodies in a self-made ELISA [32]. Mean + 2 standard deviation of optical densities (OD) measured in sera of 21 healthy age-matched controls served as cut-off for positivity. In the case of vimentin IgG, values > 1.852 OD and in the case of vimentin IgM values of > 0.790 OD were considered positive. Persons who performed the testing were blinded to all patient characteristics and outcome measures.

### Immunosuppressive regimen

The immunosuppressive regimens post-transplant and the anti-rejection therapy are described in detail in previous publications [25, 29, 33]. The initial maintenance immunosuppressive regimen consisted of a calcineurin inhibitor (CNI), either cyclosporine microemulsion (CsA) (40 of 62 (65%)) or tacrolimus (TAC) (22 of 62 (35%)), mycophenolate mofetil (100%), and methylprednisolone (100%). At time of index biopsy, three patients received the mTOR-inhibitor sirolimus without a CNI due to severe CNI-induced chronic nephrotoxicity in a previous biopsy. Twenty-three patients (37%) were treated with a glucocorticoid (steroid)-free immunosuppressive regimen. Patients with T cell-mediated allograft rejection (TCMR) of Banff type I or II were treated with methylprednisolone pulse therapy. Patients with biopsy-proven chronic ABMR received anti-humoral rejection therapy with intravenous immunoglobulin G and rituximab, as previously described [25, 33].

### Histopathology and C4d staining

Indication biopsies were performed due to rapid or progressive increase of serum creatinine (> 20% above baseline without an alternative explanation) and/or *de novo* persistent proteinuria > 100 g protein/mol creatinine and reevaluated using the Banff 2015 criteria [28]. Transplant glomerulopathy (TG) was defined as the presence of double contours by light microscopy (c.g. score > 0) in the absence of significant immune complex deposits by immunofluorescence. Immunohistochemistry for C4d was performed on paraffin sections using a polyclonal antibody (C4dpAb; Biomedica, Vienna, Austria). C4d was classified positive in the cases of focal (11 – 50%) or diffuse (> 50%) peritubular capillary C4d staining patterns.

### Statistical analysis

SPSS Statistics 22.0 (IBM, Amonk, NY, USA) and SAS 9.3 (SAS Institute, Cary, NC, USA) were used for statistical analysis. Results for continuous variables are presented as mean ± standard deviation or as median with interquartile range (IQR). Differences between groups were analyzed with one-way ANOVA, Student’s *t* test, or, if normality failed, with Kruskal–Wallis or Mann–Whitney U rank-sum test. For categorical data, Fisher exact or Pearson chi-square tests were used. Nonparametric Spearman correlation analysis was performed to analyze the relationship between non-HLA antibody values and the HLA-DSA MFI values. Receiver operating characteristics (ROC) with a bootstrapping approach (1000 repeats) were generated, and the respective area under the curves (AUC) and the 95% confidence interval (CI) limits were calculated using the method of Hanley and McNeil [34]. For survival analysis, event was defined as pronounced graft function deterioration defined as a decline of eGFR of ≥ 50% of baseline, defined as the eGFR at time of index biopsy. Graft function deterioration post-biopsy was first studied using the Kaplan–Meier method and compared using the log rank test. Separate univariate and multivariable cox regression modelling were used to quantify hazard ratios (HR) and 95% confidence intervals (CI) for clinical, immunologic, and histologic parameters at time of biopsy to event. For multivariable Cox regression models, a stepwise forward selection method was used (significance level to integrate parameters *p* < 0.1 and to remove selected parameters *p* ≥ 0.2). As there was a significant collinearity between AT_1_R and ET_A_R antibodies, the parameter “non-HLA antibody positivity” was included in the final models. Two-sided p values < 0.05 were regarded as statistically significant in a descriptive sense.

## Results

### Patient characteristics

Patient, donor, and transplant characteristics including immunosuppressive regimens at time of transplantation are depicted in Table 1, and those at time of index biopsy are given in Supplementary Table 1. The median time from transplantation to index biopsy and corresponding antibody testing was 53.5 months (IQR, 33.8 – 75.0), and patients had a median of two biopsies prior to the index biopsy. Based on the index biopsy results, patients were divided into three groups: (i) patients without rejection (*n* = 19, 30.6%), (ii) patients with features of T cell-mediated rejection (TCMR) including borderline changes (*n* = 15, 24.2%), and (iii) patients with ABMR including those suspicious for ABMR according to Banff 2015 (*n* = 28, 45.2%).

**Table 1:** Patient and transplant characteristics at time of transplantation according to antibody status at time of index biopsy

Characteristics	Entire cohort	HLA-DSA and non-HLA antibody-negative	HLA-DSA negative, non-HLA antibody-positive	HLA-DSA positive, non-HLA antibody-negative	HLA-DSA and non-HLA antibody-positive	*P* value
	(*n* = 62)	(*n* = 15)	(*n* = 18)	(*n* = 8)	(*n* = 21)	
Age (mean ± SD), years	8.6 ± 5.0	8.4 ± 4.8	9.1 ± 5.6	7.8 ± 4.7	8.8 ± 5.0	0.953
Male gender, n (%)	46 (74.2)	13 (86.7)	10 (55.6)	6 (75.0)	17 (81.0)	0.173
Donor age (mean ± SD), years	36.2 ± 12.9	31.8 ± 14.0	43.5 ± 9.8	34.8 ± 17.3	33.9 ± 11.5	0.078
Deceased donor, n (%)	40 (64.5)	11 (73.3)	11 (61.1)	5 (62.5)	13 (61.9)	0.636
Cold ischemia time (mean ± SD), hours	13.9 ± 6.4	11.9 ± 7.1	17.2 ± 4.2	17.0 ± 8.0	12.5 ± 5.2	0.412
Delayed graft function, n (%)	6 (9.7)	0 (0)	1 (5.6)	3 (37.5)	2 (9.5)	0.029
A-B-DR-DQ mismatch/8 (mea ± SD)	2.7 ± 1.2	2.7 ± 1.2	2.3 ± 1.5	3.1 ± 1.3	2.8 ± 0.8	0.388
A-B (mean ± SD)	1.3 ± 0.7	1.6 ± 0.7	1.2 ± 0.7	1.5 ± 0.8	1.2 ± 0.6	0.305
DR (mean ± SD)	0.8 ± 0.5	0.6 ± 0.6	0.7 ± 0.6	0.9 ± 0.4	0.9 ± 0.4	0.218
DQ (mean ± SD)	0.6 ± 0.6	0.5 ± 0.6	0.5 ± 0.6	0.8 ± 0.7	0.7 ± 0.6	0.549
Cause of Kidney failure, n (%)						
CAKUT	33 (53.2)	11 (73.3)	11 (61.1)	4 (50.0)	7 (33.3)	0.100
Nephronophthisis	5 (8.1)	0	3 (16.7)	0	2 (9.5)	0.275
Glomerular diseases	11 (17.7)	2 (13.3)	1 (5.6)	2 (25.0)	6 (28.6)	0.261
Others	13 (21.0)	2 (13.3)	3 (16.7)	2 (25.0)	6 (28.6)	0.673
Immunosuppression						
IL-2RA, n (%)	12 (19.4)	2 (13.3)	5 (27.8)	1 (12.5)	4 (19.1)	0.703
Cyclosporine, n (%)	40 (64.5)	8 (53.3)	8 (44.4)	8 (100)	16 (76.2)	0.022
Tacrolimus, n (%)	22 (35.5)	7 (46.7)	10 (55.6)	0	5 (23.8)	0.022
Observation time post-Tx (median, IQR), months	153 (131–185)	141 (126–162)	143 (124–184)	169 (151–190)	159 (139–190)	0.097

*DSA* donor-specific antibodies, *HLA* human leucocyte antigen, *CAKUT* congenital anomalies of the kidney and urinary tract, *IL-2RA* interleukin-2 receptor antagonist, *MMF* mycophenolate mofetil, *Tx* kidney transplantation

Baseline characteristics at time of index biopsy were overall comparable among these groups with two exceptions: patients with ABMR less frequently received a TAC-based maintenance immunosuppressive regimen than patients without rejection or patients with TCMR (50.0% vs*.* 84.2% vs. 73.3%; *p*=0.041), and the proportion of patients with proteinuria was higher in the ABMR group than in the other two groups (67.9% vs. 26.3% vs. 33.3%, *p* = 0.010, Supplementary Table 1).

### Prevalence of non-HLA antibodies in relation to HLA-DSA

**Fig. 1:**
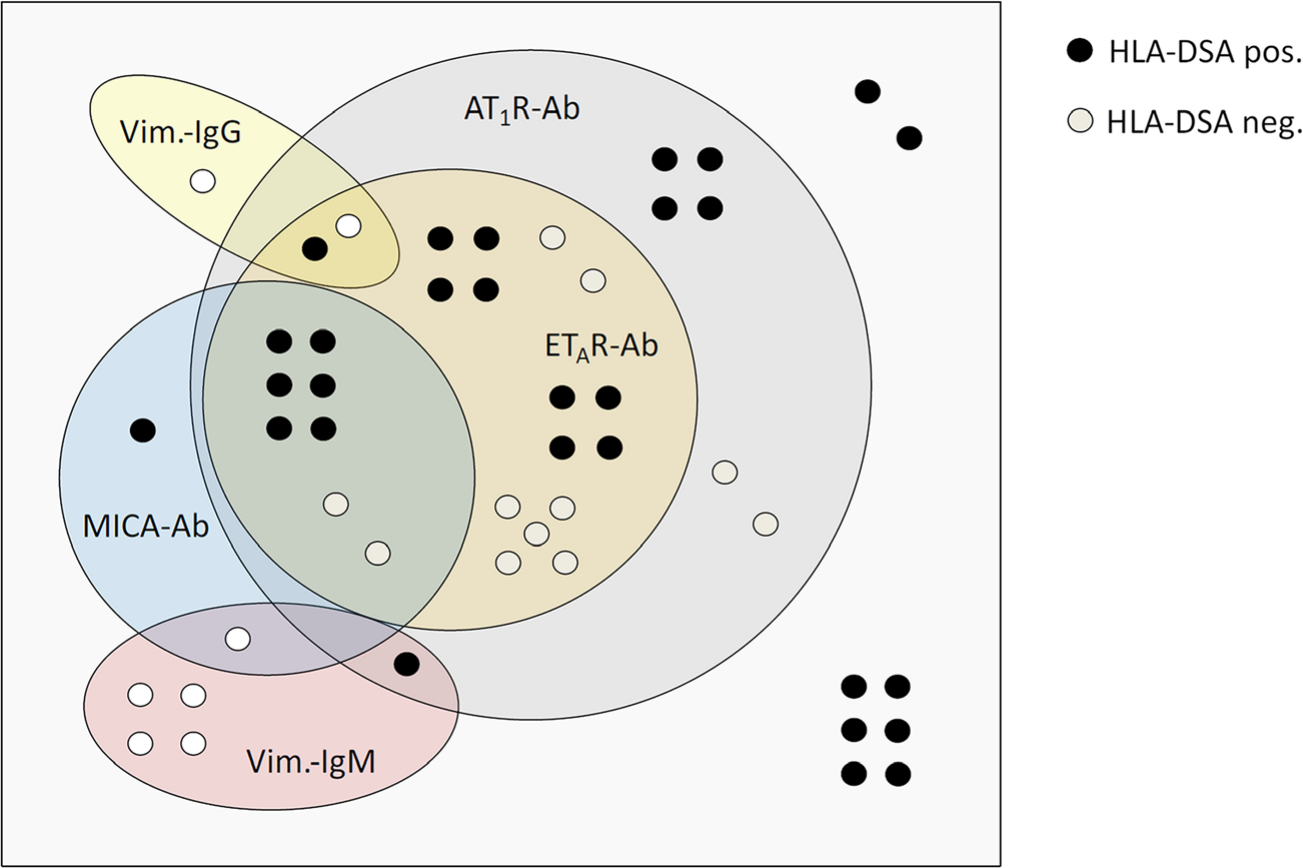
Distribution of the non-HLA antibodies (Ab) AT_1_R-Ab, ET_A_R-Ab, MICA-Ab and Vimentin-Ab stratified according to concomitant HLA-DSA positivity. AT_1_R-Ab, Angiotensin II type 1 receptor antibodies; ET_A_R-Ab, endothelin-1 type A receptor antibodies; MICA, major histocompatibility complex class I polypeptide-related sequence A; Vim., vimentin; Ab, antibodies

**Fig. 2:**
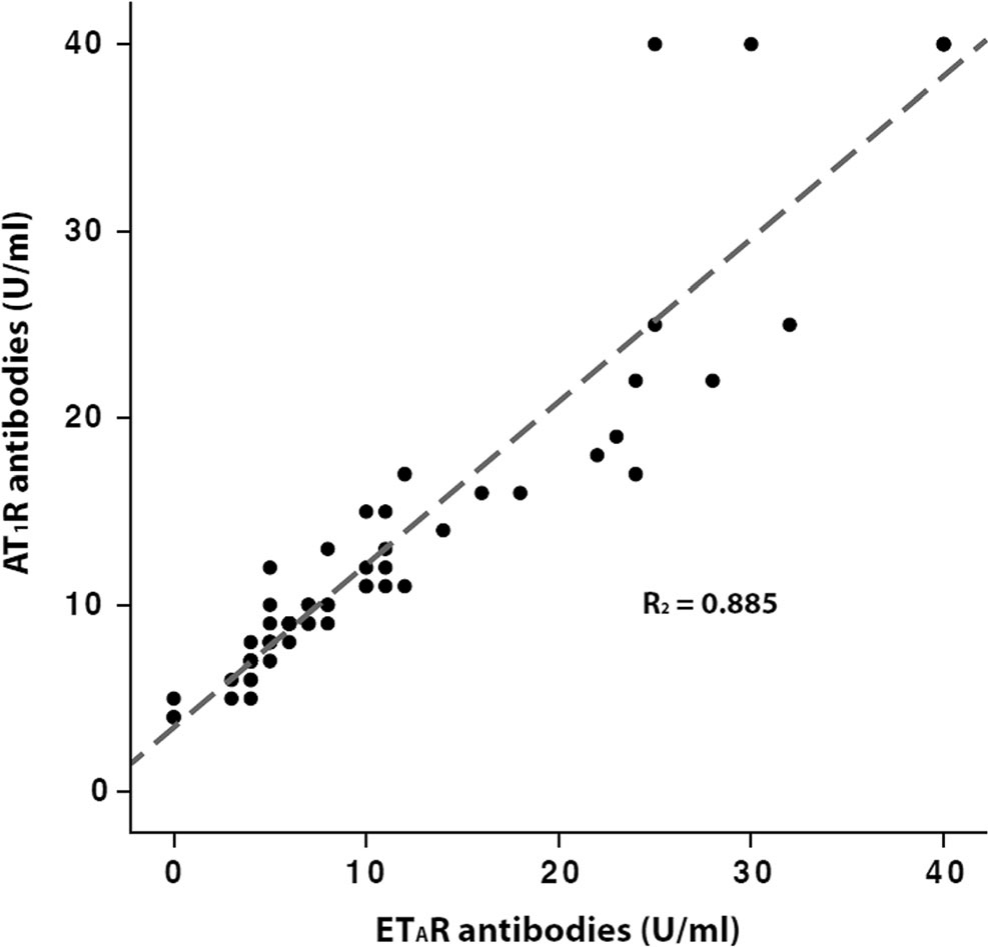
Correlation of AT_1_R-antibodies (Ab) (U/mL) and ET_A_R-Ab (U/mL) in the cohort of 62 pediatric kidney transplant recipients at the time of indication biopsy > 1-year post-transplant. AT_1_R-Ab, Angiotensin II type 1 receptor antibodies; ET_A_R-Ab, endothelin-1 type A receptor antibodies

Five different non-HLA antibodies were analyzed in conjunction with HLA-DSA. Only one patient (1.6%) showed HNA-1 antibody positivity; therefore HNA-1 antibodies were excluded from the subsequent analysis. Overall, 39 of 62 patients (62.9%) were positive for at least one non-HLA antibody. Twenty-nine (46.8%) patients were HLA-DSA positive (of these 23 (79.3%) *de novo*, three (10.3%) persistent, and three (10.3%) unknown), of whom 21 (72.4%) showed additional positivity for at least one non-HLA antibody. The number of positive non-HLA antibodies tended to be higher in HLA-DSA-positive patients (median 2.0, IQR 0 to 2.5) than in HLA-DSA-negative patients (median 1.0, IQR 0 to 2.0; *p* = 0.058). The distribution and relationship between the different non-HLA antibodies and HLA-DSA in antibody-positive patients are depicted in Fig. 1. All patients with ET_A_R antibody positivity were also AT_1_R antibody positive, while 78% of AT_1_R antibody positive patients were also ET_A_R antibody positive. A high proportion (80%) of MICA antibody-positive patients was also positive for AT_1_R and ET_A_R antibodies. Eight of 39 (20.5%) of non-HLA antibody positive patients were triple-positive for AT_1_R, ET_A_R and MICA antibodies (Fig. 1). There was a tight positive linear relationship (R^2^ = 0.885, *p* < 0.001) between ET_A_R and AT_1_R antibody values (Fig. 2). The respective number of Vimentin IgG (*n* = 3, 4.8%) and Vimentin IgM antibody (*n* = 6, 9.7%)-positive patients was low; five patients (12.8%) showed only Vimentin IgG or IgM positivity without concomitant HLA-DSA or other non-HLA antibody positivity. Overall, only 15 (24.2%) patients were negative for both HLA-DSA and non-HLA antibodies.

Table 2 shows the number (percentage) and magnitude (units) of non-HLA antibodies, stratified according to HLA-DSA status. AT_1_R and ET_A_R antibody values were significantly higher in patients with HLA-DSA positivity than in patients with HLA-DSA negativity. The proportion of AT_1_R antibody positive patients was significantly higher in the HLA-DSA positive (69%) than in the HLA-DSA negative cohort (36.4%). Also, the percentage of ET_A_R antibody positive patients tended to be higher (*p* = 0.086) in the HLA-DSA positive compared with the HLA-DSA-negative group (Table 2).

**Table 2:** Non-HLA antibody characteristics at time of index biopsy stratified according to HLA-DSA status.

Antibodies	Entire Cohort	HLA-DSA negative	HLA-DSA positive	*P v*alue*
	(*n* = 62)	(*n* = 33)	(*n* = 29)	
AT_1_R-Ab (U/mL), median (IQR)	10 (8–15)	9 (7–12)	12 (9–17)	0.013
≥ 10 U/mL; n (%)	32 (51.6)	12 (36.4)	20 (69.0)	0.010
ET_A_R-Ab (U/mL), median (IQR)	7 (5–13)	6 (4–12)	10 (6–17)	0.031
≥ 10 U/mL; n (%)	25 (40.3)	10 (30.3)	15 (51.7)	0.086
MICA-Ab (MFI), median (IQR)	151 (71–379)	134 (88–699)	174 (57–599)	0.253
MFI >500; n (%)	10 (16.1)	3 (9.1)	7 (24.1)	0.108
Vimentin IgG-Ab (OD), median (IQR)	736 (591–952)	739 (574–877)	731 (602–1016)	0.657
OD > 1.852; n (%)	3 (4.8)	2 (6.1)	1 (3.4)	0.632
Vimentin IgM-Ab (OD), median (IQR)	362 (236–490)	327 (231–550)	379 (280–481)	0.526
OD > 0.790; n (%)	6 (9.6)	5 (15.2)	1 (3.4)	0.120

^*^Significance HLA-DSA positive *vs.* HLA-DSA negative cohort. *Ab* antibodies, *HLA-DSA* human leucocyte antigen donor-specific antibodies, *AT*_*1*_*R-Ab* angiotensin receptor type 1 antibodies, *ET*_*A*_*R-Ab* endothelin-1 type A receptor antibodies, *MICA* major histocompatibility complex class I polypeptide-related sequence A, *MFI* mean fluorescence intensity, *OD* optical density

### Relationship between HLA-DSA or non-HLA antibodies and graft histology

As expected, HLA-DSA and C1q-fixing HLA-DSA were more frequent in the ABMR group than in the TCMR group or in patients without rejection (Table 3). Serum AT_1_R and ET_A_R antibody concentrations were significantly higher in the ABMR group compared with both other groups. Furthermore, the percentage of patients with AT_1_R and ET_A_R antibody positivity above the cut-off value of 10 U/mL was significantly higher in the ABMR group. This difference was no more apparent when a cut-off value for AT_1_R or ET_A_R antibody positivity of 17 U/mL was taken. As many as 26 of 28 (92.9%) patients with ABMR were either HLA-DSA (*n* = 20 (71.4%)) or AT_1_R antibody (*n* = 22 (78.6%)) positive. The remaining two patients with ABMR tested negative for any of the other non-HLA antibodies. Mean MICA antibody MFI values were significantly higher in the ABMR group than in the TCMR group. Median vimentin IgG and IgM antibody OD values were not significantly different among the three groups. More patients in the TCMR group had vimentin IgM antibodies above the cut-off value of 0.790 OD, but the overall number of patients with vimentin IgM antibodies above this threshold was low (*n* = 6) (Table 3).

**Table 3:** Antibody characteristics at time of index biopsy

Antibodies	Entire cohort	No rejection	T cell-mediated rejection*	Antibody-mediated rejection^**^	*P value*
	(*n* = 62)	(*n* = 19)	(*n* = 15)	(*n* = 28)	
HLA-DSA class I and/or II, n (%)	29 (46.8)	4 (21.1)	5 (33.3)	20 (71.4)	0.002
C1q-positive HLA-DSA	9 (14.5)	0	0	9 (32.1)	0.002
AT_1_R-Ab (U/mL), median (IQR)	10 (8–15)	8 (6–14)	9 (7–10)	12 (10–17)	0.005^#,$^
AT_1_R-Ab (≥ 10 U/mL), n (%)	32 (51.6)	5 (26.3)	5 (33.3)	22 (78.6)	0.001^#,$^
AT_1_R-Ab (> 17 U/mL), n (%)	11 (17.7)	4 (21.1)	1 (6.7)	6 (21.4)	0.435
ET_A_R-Ab (U/mL), median (IQR)	7 (5–13)	5 (4–14)	6 (4–8)	10 (6–15)	0.038^#,$^
ET_A_R-Ab (≥ 10 U/mL), n (%)	25 (40.3)	5 (26.3)	3 (20.0)	17 (60.7)	0.011^#,$^
ET_A_R-Ab (> 17 U/mL), n (%)	13 (21.0)	4 (21.1)	3 (20.0)	6 (21.4)	0.994
MICA-Ab (MFI), median (IQR)	151 (71–379)	134 (113–213)	93 (43–174)	239 (88–699)	0.048^$^
MICA-Ab (MFI > 500), n (%)	10 (16.1)	2 (10.5)	1 (6.7)	7 (25.0)	0.216
Vimentin IgG-Ab (OD), median (IQR)	736 (591–952)	671 (559–943)	701 (518–893)	785 (616–1028)	0.474
Vimentin IgG-Ab (OD > 1.852); n (%)	3 (4.8)	2 (10.5)	0	1 (3.6)	0.334
Vimentin IgM-AB (OD),median (IQR)	362 (236–490)	284 (235–367)	398 (187–794)	412 (289–491)	0.211
Vimentin IgM-Ab (OD > 0.790), n (%)	6 (9.6)	2 (10.5)	4 (26.7)	0	0.019

^*^including borderline changes; ** including suspicious for ABMR; p values are given for the overall comparision of the three groups; in addition, significant results for Bonferroni-corrected post-hoc comparisions are indicated by # for comparision between the groups ABMR and no rejection; $ for comparision between the groups ABMR and T cell-mediated rejection. *Ab* antibodies, *HLA-DSA* human leucocyte antigen donor-specific antibodies, *AT*_*1*_*R-Ab* angiotensin receptor type 1 antibodies, *ET*_*A*_*R-Ab* endothelin-1 type A receptor antibodies, *MICA* major histocompatibility complex class I polypeptide-related sequence A, *MFI* mean fluorescence intensity, *OD* optical density

**Fig. 3:**
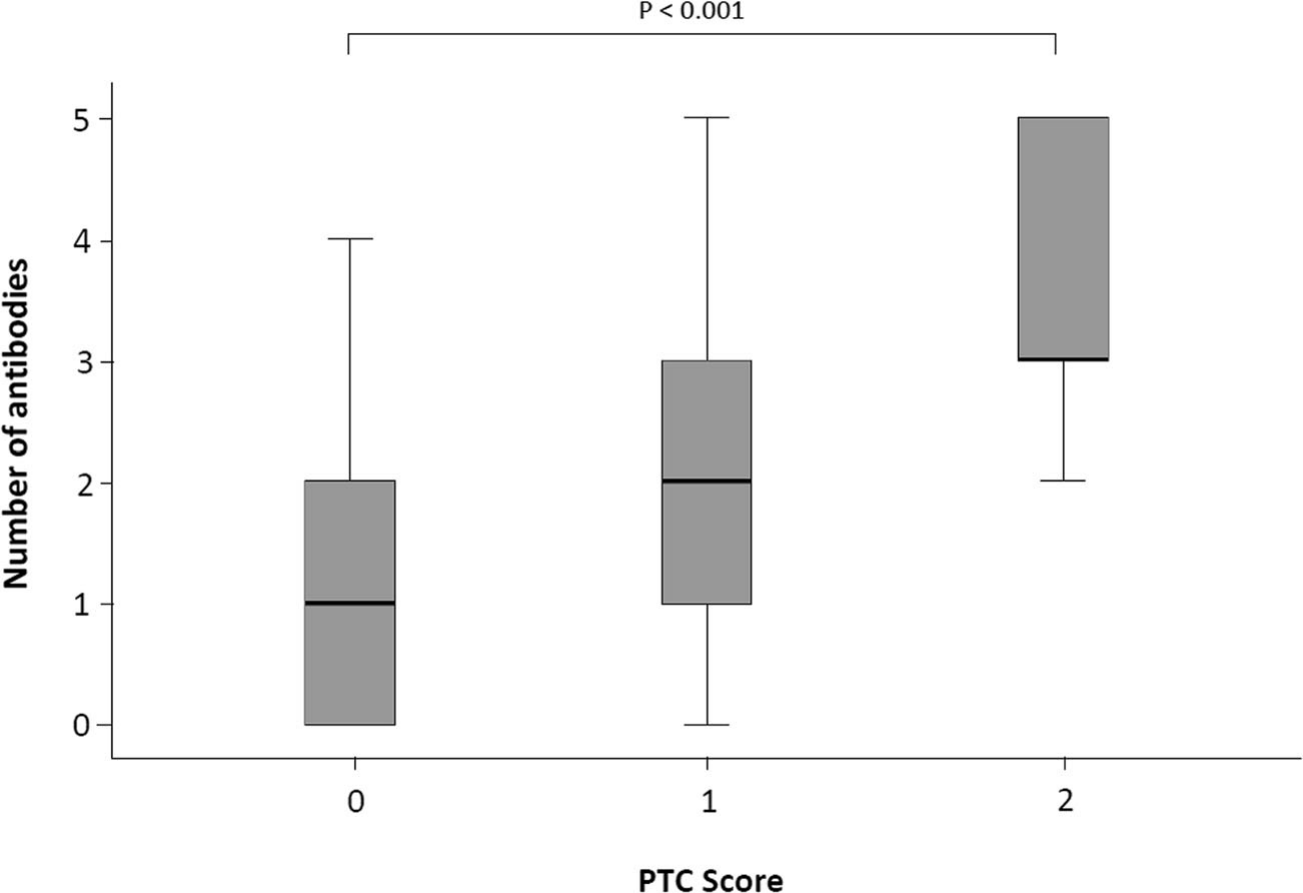
Number of HLA-DSA (class I and class II) and non-HLA antibodies (Ab) (AT_1_R-Ab, ET_A_R-Ab, MICA-Ab and Vimentin IgG, IgM) positivities per patient stratified according to the ptc status score of the index biopsy. AT_1_R-Ab, Angiotensin II type 1 receptor antibodies; ET_A_R-Ab, endothelin-1 type A receptor antibodies; MICA, major histocompatibility complex class I polypeptide-related sequence A; Ab, antibodies; ptc, peritubular capillaritis

Patients being HLA-DSA positive or double-positive for HLA-DSA and non-HLA antibodies more frequently showed peritubular capillaritis (ptc) or both ptc and glomerulitis, a hallmark of active ABMR, or chronic allograft glomerulopathy. Interestingly, the proportion of patients being double positive for HLA-DSA and non-HLA antibodies was significantly higher in the group with a ptc score of 2 compared with patients with a ptc score of 0 (100% vs. 36.8%; *p* = 0.004). The cumulative number of antibody positivities per patient was significantly higher in patients with a ptc score of 2 compared with patients with a ptc score of 0 (Fig. 3).

**Fig. 4:**
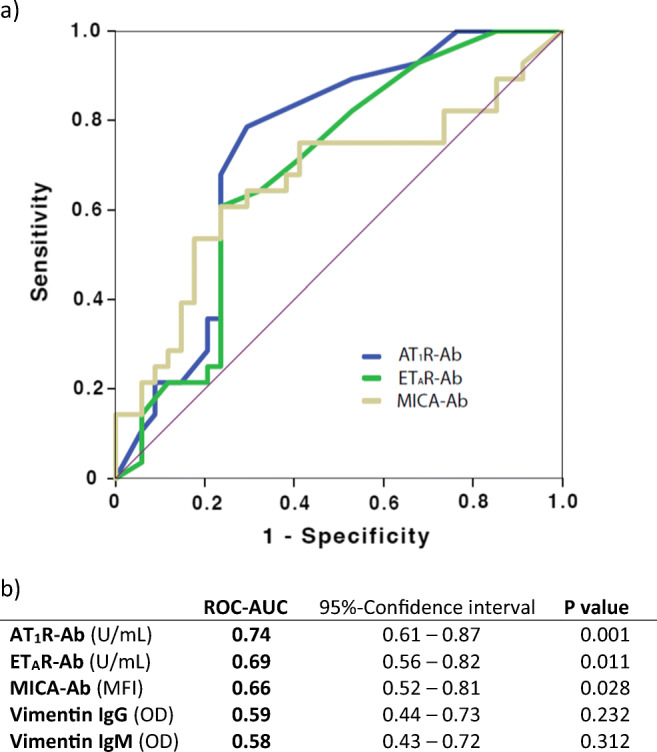
**a** ROC-curve analysis of the non-HLA antibodies concerning the discriminative capacity between antibody-mediated rejection (ABMR) and non-ABMR histopathological lesions. **b** ROC-AUC results for the different non-HLA antibodies. AT_1_R-Ab, Angiotensin II type 1 receptor antibodies; ET_A_R-Ab, endothelin-1 type A receptor antibodies; MICA-Ab, Major histocompatibility complex class I polypeptide-related sequence A antibodies; MFI, mean fluorescence intensity; OD optical density

To analyze the capacity of non-HLA antibodies for discriminating between patients with or without ABMR in the index biopsy, we performed ROC-curve analyses (Fig. 4a). Based on the ROC-AUC values, AT_1_R antibody positivity showed the best predictive capacity for differentiating between histologically confirmed ABMR and non-ABMR biopsy phenotypes, followed by ET_A_R antibody positivity and MICA antibody positivity. The discriminative capacity of vimentin IgG and IgM antibodies for ABMR was not statistically significant (Fig. 4b). Additional analyses using antibody positivity according to the respective cut-off levels as well as the summarized parameter “any positive non-HLA antibody” showed similar predictive power of the parameter “any positive non-HLA antibody” compared with the parameter “AT_1_R antibody positivity” (Supplementary Table 2).

### Risk factors for graft function deterioration

We analyzed the impact of HLA-DSA and non-HLA antibody positivity on graft function deterioration by the Kaplan–Meier log rank test. Double positivity for HLA-DSA and non-HLA antibodies was significantly associated with graft function deterioration, defined as an eGFR decline ≥ 50% of baseline, in comparison to antibody negative patients (double positivity vs*.* antibody negativity, *p* < 0.001, Kaplan-Meier log rank test; data not shown). Next, we analyzed the impact of HLA-DSA and non-HLA antibody positivity in conjunction with other non-invasive risk factors on deterioration of graft function by proportional hazard Cox regression models (Table 4A). HLA-DSA (further differentiated according to C1q-complement binding capacity), non-HLA positivity, eGFR at the time of index biopsy, proteinuria, and arterial hypertension were assessed in a multivariable Cox regression model including age at index biopsy and time period from transplantation to index biopsy (Table 4A). AT_1_R antibody positivity was significantly associated with graft function deterioration. ET_A_R antibody positivity showed a tendency in the univariate analysis (*p* = 0.081), but was not included in the multivariable model due to significant collinearity with AT_1_R antibodies (Table 4A). Introducing the parameter “non-HLA antibody positivity” in the multivariable model revealed that non-HLA antibody positivity (HR 6.38), proteinuria (HR 2.73) and eGFR at time of index biopsy were independent predictors of graft function deterioration. When histological characteristics such as transplant glomerulopathy and C4d status were added to the multivariable analysis, only transplant glomerulopathy, C1q-positive HLA-DSA, and eGFR at the time of index biopsy remained as statistically significant risk factors (Table 4B).

**Table 4:** Risk factor analysis for graft function deterioration (loss of eGFR ≥ 50% of baseline prior to index biopsy) up to five years post-biopsy

Risk factors	Unadjusted HR (95% CI)	*P Value*	Adjusted HR (95% CI)	*P Value*
**A. Serologic markers and patient characteristics**				
AT_1_R-Ab (≥ 10 U/mL)	3.14 (1.31–7.52)	0.010		
ET_A_R-Ab (≥ 10 U/mL)	1.99 (0.92–4.33)	0.081		
MICA-Ab (> 500 MFI)	1.27 (0.48–3.38)	0.628		
Non-HLA antibody positive	3.96 (1.36–11.61)	0.012	6.38 (2.11–19.29)	0.001
HLA-DSA positive	2.59 (1.15–5.83)	0.021		
Patient age at Tx, per 1-year increment	1.04 (0.96–1.13)	0.320		
Living donation	0.95 (0.42–2.12)	0.892		
Donor age, per 1-year increment	1.02 (0.98–1.05)	0.296		
Delayed graft function, per 1-day increment	1.53 (0.46–5.10)	0.493		
Proteinuria (> 20 g/mol creatinine)	3.08 (1.37–6.92)	0.007	2.73 (1.16–6.38)	0.015
eGFR at time of biopsy	0.96 (0.94–0.99)	0.017	0.96 (0.93–0.99)	0.015
Arterial hypertension	2.91 (1.22–6.96)	0.016		
**B. Including histopathological markers and complement binding HLA-DSA**				
g + ptc > 1	3.81 (1.65–8.81)	0.002		
IFTA > 2	2.05 (0.93–4.53)	0.075		
Transplant glomerulopathy	6.29 (2.83–13.99)	< 0.001	4.90 (2.01–12.03)	0.001
C4d positivity	5.10 (2.29–11.40)	< 0.001		
Non-HLA antibody positive	3.96 (1.36–11.61)	0.010		
HLA-DSA positive				
C1q negative	1.75 (0.69–4.40)	0.237		
C1q positive	6.23 (2.31–16.79)	< 0.001	4.09 (1.31–12.81)	0.015
eGFR at time of biopsy	0.96 (0.94–0.99)	0.017	0.96 (0.93–0.99)	0.013

Unadjusted HR, hazard ratio; adjusted HR, each factor has been adjusted for the other factors which have been included into the final model according to univariable p-values: HLA-DSA (further differentiated according to C1q-complement binding capacity), non-HLA antibody positivity, eGFR at the time of index biopsy, proteinuria, arterial hypertension, and in addition age at index biopsy and time period from transplantation to index biopsy; CI, confidence interval; AT_1_R-Ab, angiotensin II type 1 receptor antibodies; DSA, donor-specific HLA antibodies; C1q, C1q HLA-DSA; g, glomerulitis; ptc, peritubular capillaritis; IFTA, interstitial fibrosis and tubular atrophy; MICA, major histocompatibility complex class I polypeptide-related sequence A; Tx, transplantation; eGFR, estimated glomerular filtration rate according to Schwartz et al. [35]; Arterial hypertension, systolic or diastolic blood pressure > 1.96 SDS (95^th^. Percentile)

## Discussion

This is the first study in pediatric kidney transplant recipients that performed a comprehensive analysis of donor-specific HLA and non-HLA antibodies in relation to graft histology and function. We observed a high prevalence of non-HLA positivity: 62.9% of patients were positive for at least one non-HLA antibody. A high proportion (80%) of MICA antibody positive patients was also positive for AT_1_R and ET_A_R antibodies. Eight of 39 (20.5%) of non-HLA antibody positive patients were triple-positive for AT_1_R, ET_A_R, and MICA antibodies. The presence of these non-HLA antibodies is likely to have a functional impact on graft histology because patients being HLA-DSA positive or double-positive for HLA-DSA and non-HLA antibodies had a significantly higher vascular microinflammation score, a hallmark of active ABMR, in the graft biopsy. Furthermore, the proportion of patients with double antibody positivity, irrespective of HLA-DSA or non-HLA positivity, was significantly higher in the group of patients with pronounced ptc. Hence, there appears to be an association between the cumulative load of HLA-DSA and non-HLA antibodies in the circulation and the degree of microinflammation in peritubular capillaries of the kidney allograft. Additionally, Malheiro et al. showed in adult kidney transplant recipients a synergistic effect for pretransplant AT_1_R antibodies with HLA-DSA [36].

These data contribute to the growing evidence that specific non-HLA antibodies impact graft outcome independently and in concert with donor-specific HLA antibodies [37]. A smaller previous study in pediatric kidney transplant recipients observed that ten of 20 patients developed antibodies to one or more of the kidney-associated self-antigens AT_1_R, collagen IV, and fibronectin in the first 12 months post-transplant, but development of antibodies did not correlate with worse clinical outcomes [38].

This is the first study on post-transplant ET_A_R antibodies in pediatric kidney transplant recipients. We observed that serum ET_A_R antibody concentrations and the percentage of patients with ET_A_R antibody positivity were significantly higher in the ABMR subgroup compared with patients with T cell-mediated or no rejection. Also, in the ROC curve analysis, ET_A_R-Ab had a significant predictive capacity for differentiating between histologically confirmed ABMR and non-ABMR biopsy phenotypes. Hence, functional autoimmunity against ET_A_R is common in pediatric patients with ABMR and could contribute to disease pathogenesis. ET_A_R agonistic antibodies have been associated with other vascular diseases such as systemic sclerosis and systemic lupus erythematosus with pulmonary hypertension [39]. In adult kidney transplantation, Banasik et al. evaluated the pre-transplant presence of ET_A_R antibodies in 116 consecutive kidney transplant recipients [40]. By the use of a cut-off value of 2.5 U/mL, they observed ET_A_R antibody positivity in 47.4% of the analyzed recipients. The presence of ET_A_R antibodies was associated with a lower kidney transplant function during the subsequent 12 months post-transplant. Delville et al. investigated a highly selected cohort of 38 patients with early acute microvascular rejection (AMVR) [41]. Pre-transplant serum AT_1_R and ET_A_R antibody concentrations were not significantly increased among patients with AMVR compared with a control group of stable kidney transplant recipients. Hence, further studies are needed to precisely define the causative role of ET_A_R antibodies for ABMR.

In our study, an independent role of ET_A_R antibodies as a risk factor for graft function deterioration could not be established because of the close linear correlation between AT_1_R and ET_A_R antibodies, a finding that was reported previously in the context of kidney [41, 42] and heart transplantation [14]. Also, in patients with systemic sclerosis, the presence of AT_1_R antibodies was highly coincident with the presence of ET_A_R antibodies [43]. AT_1_R and ET_A_R antibodies have similarities to anti-endothelial cell antibodies since endothelial cells express both receptors [44], and Riemekasten et al. have shown biological effects of both antibodies in endothelial cells [43].

We observed that MICA antibodies were significantly higher in the ABMR group than in the TCMR group and had a moderate capacity for differentiating between histologically confirmed ABMR and non-ABMR phenotypes. Hence, MICA antibodies appear to play a causative or associative role in the pathogenesis of ABMR. However, MICA antibodies in our analysis were not an independent risk factor for graft function deterioration, potentially due to the small patient number or the length of follow-up. The results of other studies on the role of MICA antibodies on kidney transplant outcome are conflicting. Two large multi-center studies showed that MICA antibodies are associated with a higher incidence of allograft failure in patients without HLA antibodies or that were otherwise at low immunological risk [18, 19]. More recent but smaller studies did not show an association with unfavorable graft survival [45–47]. This could reflect that MICA antibodies are a particular risk marker for certain patient populations, while graft survival might be more strongly influenced by alternative factors in other populations.

The strength of our study is that it was based on a prospective protocol investigating a carefully phenotyped pediatric patient cohort at increased risk of graft deterioration. A limitation is the relatively small number of patients investigated, but this is an inherent problem for all studies in the pediatric kidney transplant population. Hence, the individual role of each non-HLA antibody concerning graft damage and graft function deterioration has to be clarified in larger prospective studies, ideally by utilizing an even broader non-HLA antibody panel. Non-HLA antibodies were measured retrospectively only at a single time point post-transplant; we therefore cannot differentiate between preformed and *de novo* non-HLA antibodies. In addition, the association between non-HLA antibodies and HLA-DSA might be overestimated because of the single point measurement in the DSA-positive indication biopsy group. Furthermore, according to the study design, we cannot extrapolate the results to biopsies earlier than 1-year post-transplant. Also, the impact of subclinical rejection and of only intermittent appearance of some DSA on the development of ABMR might be undervalued. Also, groups which where HLA-DSA-positive had roughly 2 more years follow-up than the antibody-negative group. Future studies will have to evaluate whether longitudinal analyses of these antibodies in an unselected patient cohort of pediatric kidney allograft recipients is a surrogate marker for increased immunological risk requiring more intense immunosuppressive therapy. It will also be important to investigate the effect of potential treatment modalities on non-HLA antibodies and if they improve outcomes for these patients.

In conclusion, we observed a high prevalence (62.9%) of non-HLA antibody positivity in a pediatric patient cohort at increased risk of graft deterioration. Seventy-two percent of HLA-DSA-positive patients showed additional positivity for at least one non-HLA antibody. Antibodies against AT_1_R, ET_A_R, and MICA were associated with the histological phenotype of ABMR. Non-HLA antibody positivity was an independent non-invasive risk factor for graft function deterioration, whereby AT_1_R antibodies had the highest impact on graft damage and graft function deterioration in this cohort. Hence, the combined detection of antibodies to HLA and non-HLA targets may allow a more comprehensive assessment of the patients’ immune responses against the kidney allograft and will facilitate immunological risk stratification. Hopefully, targeted enhancement of immunosuppressive therapy in patients at high risk for ABMR will improve transplant outcome.

## Supplementary Information

ESM 1 (DOCX 54 kb).

## Data Availability

From the corresponding author
