# Supplementary material for: Association of non-HLA antibodies against endothelial targets and donor-specific HLA antibodies with antibody-mediated rejection and graft function in pediatric kidney transplant recipients
Source: Pediatr Nephrol. 2021 Mar 24;36(8):2473–84. doi: 10.1007/s00467-021-04969-1 (PMC8260519; doi:10.1007/s00467-021-04969-1)
Supplement: Supplementary file 1 — (DOCX 54 kb). [file 467_2021_4969_MOESM1_ESM.docx]

**Supplementary Information**

**Supplementary Table 1. Patient and transplant characteristics at time of index biopsy.**

| **Characteristics** | **Entire**  **Cohort** | **No rejection** | **T-cell mediated rejection^*^** | **Antibody- mediated rejection^**^** | ***P-Value*** |
| --- | --- | --- | --- | --- | --- |
|  | (n = 62) | (n = 19) | (n = 15) | (n = 28) |  |
| Age (mean ± SD), years | 13.7 ± 5.5 | 13.3 ± 5.2 | 12.5 ± 5.4 | 14.5 ± 5.7 | 0.489 |
| eGFR (mean ± SD), mL/min x 1.73 m^2^ | 41.0 ± 15.3 | 44.6 ± 16.8 | 42.8 ± 12.0 | 37.5 ± 15.5 | 0.260 |
| Proteinuria, n (%), (> 100 g/mol creatinine) | 8 (12.9) | 2 (10.5) | 0 | 6 (21.4) | 0.127 |
| Proteinuria, n (%), (>20 g/mol creatinine) | 29 (46.8) | 5 (26.3) | 5 (33.3) | 19 (67.9) | 0.010 |
| Number of pre-biopsies per patient (median, IQR) | 2.0 (0 – 4.0) | 2.0 (1.0 – 3.0) | 2.0 (1.0 – 2.0) | 2.0 (0.25 – 3.0) | 0.943 |
| Patients with BPAR (≥ BANFF IA) in pre-biopsies, n (%) | 17 (27.4) | 4.0 (21.1) | 3 (20.0) | 10 (35.7) | 0.413 |
| Patients with treated AR in pre-biopsies, n (%) | 33 (53.2) | 13 (68.4) | 6 (40.0) | 14 (50.0) | 0.231 |
| Immunosuppression  Tacrolimus, n (%)  Cyclosporine, n (%)  CNI-free/SRL, n (%)  Prednisolone, n (%)  MMF, n (%) | 41 (66.1)  18 (29.0)  3 (4.8)  39 (62.9)  52 (83.9) | 16 (84.2)  3 (15.8)  0  12 (63.2)  16 (84.2) | 11 (73.3)  4 (26.7)  0  13 (86.7)  11 (73.3) | 14 (50.0)  11 (39.3)  3 (10.7)  14 (50.0)  25 (89.3) | 0.041  0.214  0.147  0.060  0.399 |
| A-B-DR-DQ mismatches/8 | 2.7 ± 1.2 | 2.6 ± 1.7 | 2.5 ± 0.8 | 2.9 ± 1.0 | 0.558 |
| AB, (mean ± SD) | 1.3 ± 0.7 | 1.5 ± 0.9 | 1.3 ± 0.5 | 1.3 ± 0.7 | 0.568 |
| DR, (mean ± SD) | 0.8 ± 0.5 | 0.6 ± 0.7 | 0.7 ± 0.5 | 0.9 ± 0.4 | 0.138 |
| DQ, (mean ± SD) | 0.6 ± 0.6 | 0.5 ± 0.7 | 0.4 ± 0.6 | 0.7 ± 0.5 | 0.255 |
| Patients with HLA-DSA  Class I, n (%)  Class II, n (%) | 29 (46.8)  12 (19.4)  27 (43.5) | 4 (21.1)  1 (5.3)  3 (15.8) | 5 (33.3)  0  5 (33.3) | 20 (71.4)  11 (29.3)  19 (67.9) | 0.002  0.001  0.001 |
| Time Tx to biopsy (median, IQR), months | 53.5 (33.8 – 75.0) | 51.0 (27.0 – 63.0) | 35.0 (24.0 – 60.0) | 62.0 (39.3 – 109.0) | 0.067 |
| Follow-up post-biopsy (median, IQR), months | 79.5 (65.0 – 99.0) | 73.0 (62.0 – 88.0) | 90.0 (69.0 – 105.0) | 80.5 (63.5 – 115.0) | 0.390 |

^*^ including borderline changes; ** including suspicious for ABMR; eGFR, estimated glomerular filtration rate according to Schwartz et al. [37]; BPAR, biopsy-proven acute rejection; AR, acute rejection; TG, transplant glomerulopathy; CNI, calcineurin inhibitor; mTOR, mechanistic target of rapamycin; MMF, mycophenolate mofetil; HLA-DSA, human leucocyte antigen donor specific antibodies; SRL, sirolimus; Tx, kidney transplantation

**Supplementary Table 2. ROC-AUC analysis based on non-HLA antibody positivity for prediction of ABMR and for prediction of graft function deterioration.**

|  | **ROC-AUC** (prediction of ABMR) | P value | **ROC-AUC** (prediction of graft function deterioration) | P value |
| --- | --- | --- | --- | --- |
| AT_1_R-Ab (≥ 10 U/mL) | 0.746 | 0.001 | 0.685 | 0.012 |
| ET_A_R-Ab (≥ 10 U/mL) | 0.686 | 0.012 | 0.616 | 0.120 |
| MICA (MFI > 500) | 0.581 | 0.276 | 0.527 | 0.721 |
|  |  |  |  |  |
| Non-HLA antibody positive (AT_1_R, ET_A_R, MICA^*^) | 0.749 | 0.001 | 0.690 | 0.011 |

ABMR, antibody mediated rejection; graft deterioration defined as loss of eGFR ≥ 50% of baseline prior to index biopsy up to 5 years post-biopsy; AT_1_R-Ab, Angiotensin II type 1 receptor antibodies; ET_A_R-Ab, endothelin-1 type A receptor antibodies; MICA, major histocompatibility complex class I polypeptide-related sequence A; Ab, antibodies; ^*^ any of the non-HLA antibodies (AT_1_R, ET_A_R, MICA) positive

**Supplementary Figure 1:**

Patients excluded due to:

1. presensitization (n = 9)
2. ABO-incompatible transplantation (n = 1)
3. patients with combined transplants (n = 3)
4. regular post-transplant follow-up in other institutions (n = 19)

Patients without late indication biopsies (> one year post-transplant) or late indication biopsies, but at least one late biopsy before 2004

n = 39

Patients with late indication biopsy
(> one year post-transplant)

1. Insufficient Serum
2. Loss to follow-up

n = 8

**Final cohort**

n = 62

Patients with at least one late indication biopsy (> 1 year post-transplant)

n = 70

Patients fulfilling the baseline inclusion criteria

n = 109

Patients transplanted between January 1, 1999 and January 31, 2010 in our institution (University Children’s Hospital, Heidelberg)

n = 141

Patients with one late indication biopsy

n = 30

Patients with more than one late indication biopsy

n = 32

- One previous late indication biopsy before “index biopsy “ (n = 20)
- More than one previous late indication biopsy before “index biopsy” (n = 12)
